# An investigation of the auto-induction of and gender-related variability in the pharmacokinetics of dihydroartemisinin in the rat

**DOI:** 10.1186/1475-2875-11-379

**Published:** 2012-11-21

**Authors:** Fanping Zhu, Fuying Du, Xinxiu Li, Jie Xing

**Affiliations:** 1 School of Pharmaceutical Sciences, Shandong University, 44# West Wenhua Road, Jinan, 250012, People’s Republic of China

**Keywords:** Dihydroartemisinin, Metabolites, Autoinduction, Sex difference

## Abstract

**Background:**

Artemisinin (QHS) and its derivatives dihydroartemisinin (DHA), artemether and artesunate have become the first-line anti-malarials in areas of multidrug resistance. Declining plasma concentrations during the repeated dosing have been reported for QHS, artemether and less convincingly for artesunate (ARS). However, there is limited information on whether the concentrations of their active metabolite DHA and its subsequent metabolites increased after multiple drug administrations. This study was designed to evaluate the potential auto-induction metabolism of DHA in animal species. The sex-specific effect on the pharmacokinetic profiles of DHA and its metabolites was studied. The pharmacokinetics of ARS, the prodrug of DHA, and its phase I/II metabolites were also investigated.

**Methods:**

Two groups of rats received a single oral dose of DHA or ARS, and another two groups of rats were given oral doses of DHA or ARS once daily for five consecutive days. Plasma samples were analyzed for DHA, ARS and their phase I/II metabolites, using a validated liquid chromatography tandem mass spectrometric (LC-MS) method.

**Results:**

DHA, monohydroxylated DHA (M1) and the glucuronide of DHA (DHA-G) were detected in rat plasma after oral administration of DHA or ARS. Neither DHA nor its metabolites (M1 and DHA-G) changed significantly (*P* > 0.05) in AUC_*0*-*t*_ after 5-day oral doses of DHA or ARS. Sex difference was observed for DHA and its metabolites (M1 and DHA-G), whereas its prodrug ARS did not show similar characteristics for the corresponding metabolites (DHA, M1 and DHA-G).

**Conclusions:**

The results gave the direct evidence for the absence of auto-induction of phase I and phase II metabolism of DHA and ARS in rats. The sex effect existed for DHA but not for ARS, which could be caused by the sex-specific differences in absorption of DHA.

## Background

**Figure 1:**
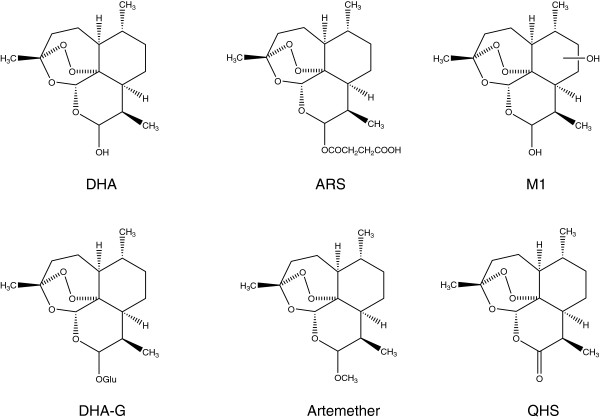
Structures of dihydroartemisinin (DHA), artesunate (ARS), monohydroxylated DHA (M1), the glucuronide of DHA (DHA-G), artemether, and artemisinin (QHS).

Dihydroartemisinin (DHA, Figure 1) is a semisynthetic anti-malarial derivative of artemisinin (Qing-hao-su, QHS). It is widely used currently in the clinic for the treatment of uncomplicated, complicated, or severe malarias, including multidrug-resistant falciparum malaria [1]. Artemisinin-based combination therapy (ACT) is the recommended treatment for uncomplicated *Plasmodium falciparum* malaria by WHO, and DHA-piperaquine (Artekin®) has been proven to be a prospective candidate for ACT [2]. DHA is also the common metabolite of its methyl ether (artemether) and hemisuccinate ester artesunate (ARS), both of which are used in the treatment of malaria [3,4]. ARS could be rapidly converted to DHA (t_1/2_, 2-3 min), which in turn was eliminated from the systemic circulation with a t_1/2_ of 40-50 min [5]. Thus, most of the anti-malarial activity resulting from ARS administration is thought to be attributable to DHA. Thirteen phase I metabolites and three phase II metabolites of DHA have been detected in human liver microsomes and rat liver microsomes [6]. Metabolism of DHA was observed with high excretion via bile into intestines and approximately 89-95% dose of all conjugates were accounted for in rat blood, urine and faeces [7]. The phase II metabolite α-DHA-G is an important metabolite of DHA in humans, and its formation is catalyzed by UGT1A9 and UGT2B7 [5]. More importantly, the glucuronide adducts possess high-to-moderate anti-malarial activity [8].

QHS and artemether exhibited obvious time-dependent pharmacokinetics in humans, and their plasma concentrations decreased after multiple oral doses [9,10]. Induction of the CYP2B6 and CYP3A4, which are involved in the metabolism of artemisinin, has been implicated as the underlying mechanism of their time dependent pharmacokinetics [11,12]. A 5-day multiple dosing study of ARS in patients showed less convincing evidence, in which the plasma concentration of DHA at 2 h after each oral dose showed a time-dependent decline [13]. However, another study did not show time-dependent pharmacokinetics of ARS and its metabolite DHA in healthy subjects [14]. A pharmacokinetic study on DHA in patients showed no differences in the concentrations of DHA within the first two days of treatment, whereas significant lower AUC of DHA was observed on the fifth day, which was implicated to be the results of physiological changes and possible auto-induction metabolism [15].

Although, the disposition of artemisinin drugs has been well studied in rodents and humans, no direct evidence was available for the increased concentrations of phase I/II metabolites of DHA in blood circulation system after repeated administrations, partly because of the extensive metabolism of DHA and the unavailability of standards. The analysis of phase I/II metabolites of DHA will be important to understand the auto-induction of these drugs, since DHA is the major active metabolite of artemisinin drugs and a preliminary investigation of DHA (as well as ARS) displayed less convincingly time-dependent pharmacokinetics.

Moreover, sex-specific differences in pharmacokinetics have been reported to have important clinical consequences, and it could become a predominant determinant of inter-individual differences in therapeutic responses [16]. Some representative sex-specific differences exist in absorption and transporters (e.g. P-glycoprotein), metabolic and clearance processes, and involvement of sex hormones (i.e.,oestrogen and testosterone) in the regulation of some metabolic enzymes. Thus, studies of sex-specific differences in pharmacokinetics of DHA and its metabolites would be important to understand the potential auto-induction metabolism of DHA. The pharmacokinetic properties of DHA were found to be affected by gender and body weight in malarial patients [17], whereas the gender difference was not observed in healthy volunteers [18].

Based on the metabolite identification of DHA, the pharmacokinetic study of DHA and its phase I/II metabolites in rats after a single and multiple oral doses of DHA (as well as its prodrug ARS) was performed in the present work. The sex difference was also evaluated.

## Methods

### Chemicals and reagents

DHA, ARS and QHS (as internal standard) were provided by Kunming Pharmaceutical Co. (purity >99.0%, Yunnan, China). DHA glucuronide (DHA-G, Figure 1) was synthesized, and its structure was confirmed by HR-MS, ^1^H-NMR and ^13^C-NMR spectroscopy. Acetonitrile and methanol (HPLC grade) were purchased from Fisher Chemicals (Fairlawn, NJ, USA). All other chemicals used were purchased from Sigma-Aldrich or Thermo Fisher Scientific, and were of highest purity available.

### Instrumentation

All LC-MS experiments were carried out on a Thermo Electron LTQ-Orbitrap XL hybrid mass spectrometer (ThermoFinnigan, Bremen, Germany) equipped with an electrospray ionization interface. An Accela HPLC system (ThermoElectron) was equipped with an autosampler, a vacuum degasser unit, and a quaternary pump. The mass spectrometer employing positive ionization was calibrated using the manufacturer’s calibration standards mixture allowing for mass accuracies <5 ppm in external calibration mode. The ionization voltage was 4.2 kV and the capillary temperature was set at 275°C. Nitrogen was used as both the sheath gas (40 units) and auxiliary gas (10 units). The full scan mode across an *m*/*z* range that bracketed DHA and its potential phase I/II metabolites was used. Extracted ion chromatograms (EICs) of *m*/*z* 302.196 for DHA, *m*/*z* 318.191 for monohydroxylated DHA (M1), *m*/*z* 478.228 for DHA-G, *m*/*z* 402.212 for ARS, and *m*/*z* 283.154 for QHS, the internal standard, with a 10 ppm range centered on the exact *m*/*z* value were generated for quantitation.

Chromatographic separation was achieved on a Luna ODS C18 column (150×2.1 mm i.d., 5 μm; Phenomenex, Torrance, CA) with a 4.0×3.0 mm i.d. SecurityGuard C18 (5 μm) guard column (Phenomenex, Torrance, CA). The chromatography was performed at 22°C. The mobile phase consisted of acetonitrile/methanol/5 mM aqueous ammonium acetate containing 0.05% (v/v) formic acid (55:30:15, v/v), delivered isocratically at a flow rate of 0.35 mL/min for quantitation assays of ARS, DHA and their metabolites. The autosampler was set at 4°C.

### Quantification of ARS, DHA and their metabolites in rat plasma

Plasma samples were subjected to a protein precipitation extraction process, which was performed on ice. A 25 μL aliquot of rat plasma was mixed with 100 μL of IS (4 μM, prepared in methanol) and 5 μL of methanol. The mixture was mixed and centrifuged at 3000 g for 10 min. Aliquots (20 μL) of the solution were injected onto LC-MS analysis. For calibration preparation, 25 μL of drug-free plasma was mixed with 5 μL of stock solution (ARS, DHA and DHA-G) and 100 μL of IS. This mixture was treated as above. The calibration graph was plotted by least-squares linear regression of the peak-area ratios (ARS, DHA or DHA-G to internal standard) against concentrations of ARS, DHA or DHA-G. Matrix matched calibration standards were obtained with concentrations of 20-2000 nM for DHA-G and 10-1000 nM for ARS and DHA in plasma. QC samples were obtained with three concentration levels (40, 400 and 1600 nM for DHA-G; 20, 200 and 800 nM for ARS and DHA) in plasma. Plasma samples were diluted with blank plasma and reanalyzed when the concentration of DHA was higher than the upper limit of quantification.

Quantitative data of the monohydroxylated metabolite of DHA (M1) was extracted based on the calibration curve of DHA. In this study, the pharmacokinetic profile of M1 in rats after a single oral dose of DHA or ARS will be used to compare with that after a multiple oral dose, in order to evaluate the auto-induction metabolism. In this case, the response factor for M1 compared with that of DHA was not considered.

### Method validation

The method was evaluated through linearity, intra- and inter-day precision and accuracy. The accuracy and precision of the method were assessed by determining QC samples using six replicated preparations of plasma samples at three concentration levels (40, 400 and 1600 nM for DHA-G; 20, 200 and 800 nM for ARS and DHA) on three separate days. The LLOQ represents the lowest concentration of the analyte over the linear range that can be determined with acceptable precision and accuracy.

Bench-top stability was assessed by leaving the QC samples of two different concentrations (40 and 1600 nM for DHA-G; 20 and 800 nM for ARS and DHA) on ice for 2 hours. The stability of samples in autosampler vials was assessed at 4°C for 8 hours.

### Drug administration and sample collection

Wistar rats (230-250 g) were supplied by Laboratory Animal Center of Shandong University (Grade II, Certificate No. SYXK 2003-0004). The experimental protocol was approved by the University Ethics Committee and conformed to the “Principles of Laboratory Animal Care” (NIH publication no. 85-23, revised 1985). Rats were maintained at 22 ± 2 °C and 55 ± 5% relative humidity on a 12 h light–dark cycle for at least 5 days before being used. Rats were fasted for 12 h before drug administration and for a further 3 h after dosing. Water was freely available for rats during experiments. A group of male rats (n = 6) and a group of female rats (n = 6) were given a single oral dose of DHA or ARS 10 mg/kg. Another group of male rats (n = 6) and female rats (n = 6) were orally given DHA or ARS 10 mg/kg once daily for five consecutive days. DHA was suspended in soybean oil, and ARS was dissolved in sodium bicarbonate injection (5%, pH 8.9). After oral administration, blood samples of 150 μL were withdrawn from the jugular vein before dosing and at 0.08, 0.17, 0.33, 0.5, 0.67, 1.0, 1.5, 2.0, 2.5, 3.0, 4.0 and 5.0 h post-dosing. All heparinized blood samples were centrifuged at 3,000 g for 15 min and plasma was obtained. All the plasma samples were stored at -80°C and assayed within one month.

### Data processing

The peak plasma concentration (*C*_max_) and time-to-peak concentration (*T*_max_) were obtained from experimental observations. The other pharmacokinetic parameters were analysed by noncompartmental model using the program TOPFIT (version 2.0; Thomae GmbH, Germany). The area under the plasma concentration-time curve (AUC_*o*-*t*_) was calculated using the linear trapezoidal rule to the last point. The mean residence time (MRT) was obtained by dividing the area under the first moment-time curve (AUMC_*0*-*t*_) by the area under the curve (AUC_*0*-*t*_). Total oral body clearance (CL/F) was calculated as dose/AUC_*0*-*t*_.

Results were expressed as mean ± SD. The two-tailed *t*-test was used for independent sample comparison of the parameters between the single dose and multiple dose groups. The acceptable level of significance was established at *P* < 0.05.

## Results and discussion

### Metabolite identification using LTQ/Orbitrap

**Figure 2:**
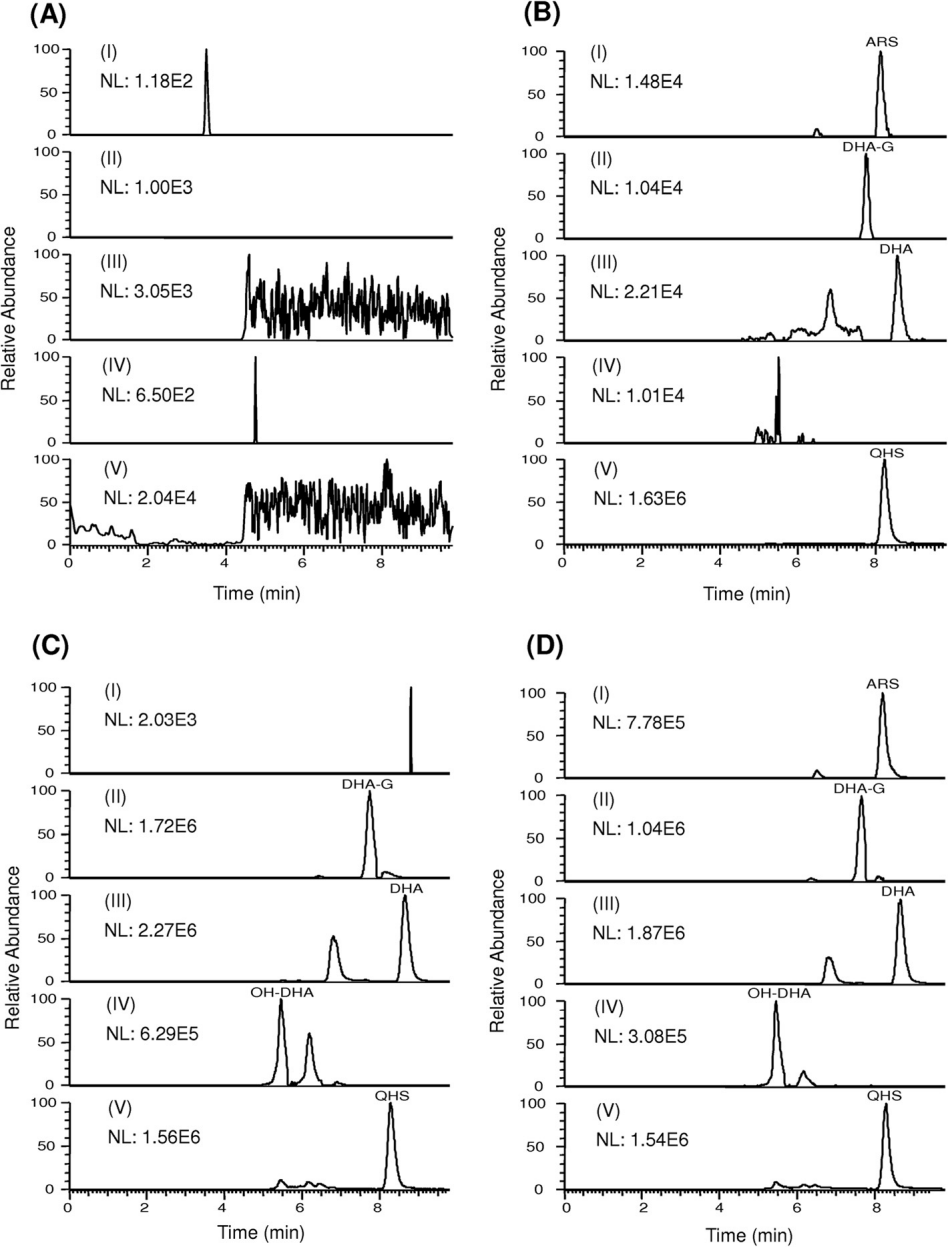
**Representative full-scan chromatograms of (A) a blank rat plasma sample; (B) a blank rat plasma sample spiked with dihydroartemisinin (DHA; 10 nM), artesunate (ARS; 10 nM), the glucuronide of DHA (DHA-G; 20 nM) and IS (QHS, 4 μM); (C) a rat plasma sample at 0.17 h after a single oral dose of DHA (10 mg/kg); and (D) a rat plasma sample at 0.17 h after a single oral dose of ARS (10 mg/kg). I: ARS (*m/z*402.2122); II: the glucuronide of DHA (*m/z*478.2283); III: DHA (*m/z*302.1962); IV: the monohydroxylated metabolite of DHA (M1;*m/z*318.1911); V: IS (*m/z*283.1540).**

Rat plasma samples of DHA were analysed by LC-HR/MS, as illustrated in selected ion chromatograms of DHA, its phase I metabolite (M1) and phase II metabolite DHA-G (Figure 2). Based on the accurate MS and MS/MS spectra, M1 was proposed to be monohydroxylated DHA. Their proposed structures are shown in Figure 1. The metabolic profile of ARS, the prodrug of DHA, was similar to that of DHA in rats. The identification of phase I and II metabolites of DHA *in vitro* and *in vivo* using LTQ/Orbitrap has been shown in detail in a previous study [6]. In the present study, the pharmacokinetics of DHA and its two metabolites (M1 and DHA-G) in relatively high concentration level were studied.

## LC-MS method

Under optimized HPLC conditions, DHA, ARS and their phase I/II metabolites were eluted within 10 min (Figure 2). Blank rat plasma from six lots showed no significant interfering peaks at the retention times of each analyte (Figure 2A). The calibration curves of ARS, DHA and DHA-G were linear over the concentration range of 10-1000 nM, 10-1000 nM and 20-2000 nM, respectively, in rat plasma with correlation coefficients *r* > 0.99 when evaluated by weighed (1/*x*^*2*^) least-squares linear regression. The lower limits of quantification (LLOQ) of ARS, DHA and DHA-G in rat plasma were established at 10, 10 and 20 nM, respectively (Figure 2B). The precision and accuracy of this method indicate that all coefficients of variation at each concentration level are below 15%. Previous studies have shown that ARS and DHA are not stable at room temperature [19], and plasma samples were processed on ice and stored at low temperature in the present study. There was no significant difference (<15%) between the responses of standards at time zero and after storage of plasma on ice for at least 2 hours in terms of %CV for ARS (6.8%), DHA (3.8%) and DHA-G (6.1%), indicating that they were stable under this condition. Processed samples were stable (%CV <15%) for at least 8 hours in the autosampler tray.

 The semi-quantitation of the monohydroxylated metabolite of DHA (M1) was performed in this study due to unavailability of the standard. The pharmacokinetic profiles of DHA and its metabolites in rats after a single oral dose will be used to compare with that after a multiple oral dose, in order to evaluate the auto-induction metabolism. Based on past experience it is reasonable to expect that M1 will exhibit similar ionization efficiency to the parent molecule DHA in positive ion mode. In this case, the response factor for M1 compared with that of DHA was not considered, and quantitative data of M1 could be extracted based on the calibration curve of DHA.

### Pharmacokinetics of DHA and its phase I/II metabolites

**Figure 3:**
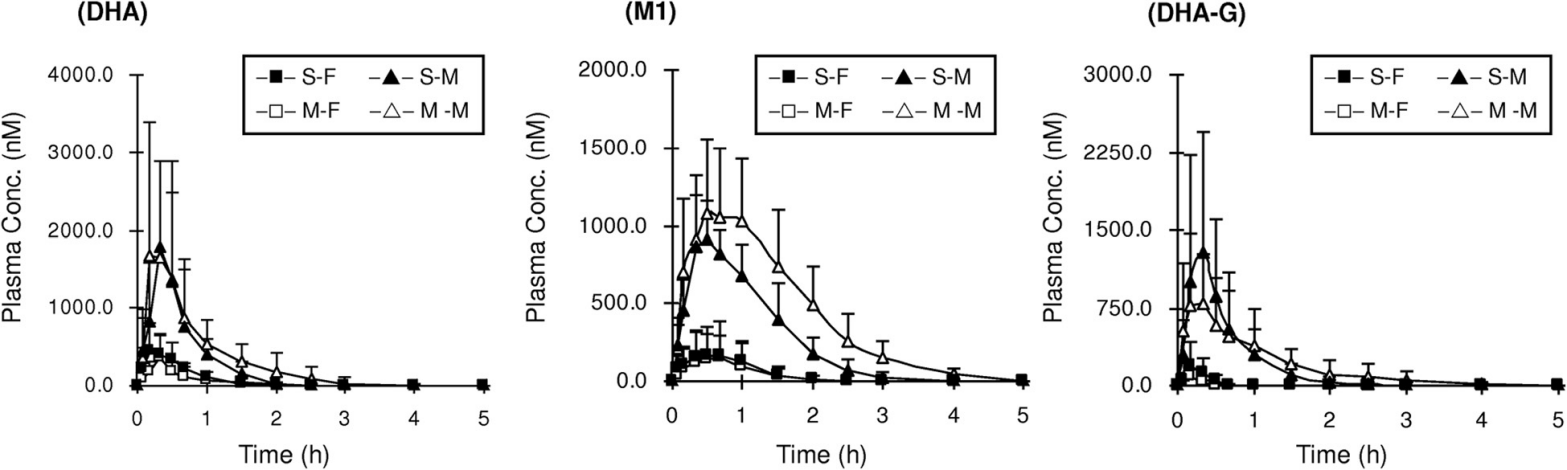
Mean (+S.D.) plasma concentration-time profiles of dihydroartemisinin (DHA), the monohydroxylated DHA (M1) and the glucuronide of DHA (DHA-G) in male (triangular points; S-M and M-M) and female (square points; S-F and M-F) rats (n = 6 each) following a single oral administration (solid points) or five consecutive days of oral doses (open points) of DHA (10 mg/kg).

After DHA was orally administered to rats, DHA and its two major phase I (M1) and phase II (DHA-G) metabolites could be detected. The major metabolic pathways are hydroxylation and glucuronidation. Their mean plasma concentration-time profiles of DHA and its phase I/II metabolites after oral dosing of DHA are shown in Figure 3, and the pharmacokinetic parameters are given in Table 1.

**Table 1:** The main pharmacokinetic parameters of dihydroartemisinin (DHA) and its phase I/II metabolites (M1 and DHA-G) after one-day and five-day oral administrations of DHA (10 mg/kg) to male (M) and female (F) rats (mean ± s.d)

			**AUC**_**0-t**_**(nmol⋅h⋅L**^**-1**^**)**	**C**_**max**_**(nmol⋅L**^**-1**^**)**	**T**_**max**_**(h)**	**MRT (h)**	**CL/F (L/min/kg)**
One-day	DHA	M	1110.35 ± 724.56†	2062.22 ± 1364.13†	0.3 (0.2-0.5)	0.6 ± 0.1	0.75 ± 0.45†
		F	322.16± 159.67†	562.71 ± 273.86†	0.3 (0.2-0.5)	0.6 ± 0.1	2.36 ± 1.40†
	M1	M	1202.13± 418.50†	996.67 ± 244.81†	0.4 (0.3-0.5)†	1.0 ± 0.1†	0.53 ± 0.14†
		F	191.46 ± 189.95†	180.02 ± 158.28†	0.5 (0.3-0.7)†	0.7 ± 0.1†	5.48 ± 3.98†
	DHA-G	M	855.69 ± 789.17†	1443.21 ± 1222.24†	0.3 (0.2-0.5)	0.6 ± 0.2†	1.78 ± 1.98†
		F	58.16 ± 40.88†	236.11 ± 232.63†	0.2 (0.2-0.3)	0.3 ± 0.1†	10.43 ± 5.63*†
Five-day	DHA	M	1512.55 ± 950.76†	2605.58 ± 1677.94†	0.4 (0.2-0.7)	0.8 ± 0.3	0.72 ± 0.82†
		F	212.63 ± 202.33†	378.05 ± 289.44†	0.3 (0.2-0.3)	0.6 ± 0.1	4.43 ± 2.83†
	M1	M	2034.92± 903.13†	1102.67 ± 478.42†	0.5 (0.3-0.7)	1.3 ± 0.2†	0.35 ± 0.19†
		F	157.11 ± 249.88†	153.58 ± 229.01†	0.6 (0.5-0.7)	0.7 ± 0.1†	8.77 ± 6.61†
	DHA-G	M	907.28 ± 542.38†	1155.90 ± 609.47†	0.3 (0.2-0.7)	1.0 ± 0.6†	0.82 ± 0.39†
		F	19.33 ± 15.27†	125.30 ± 58.48†	0.2 (0.2-0.2)	0.2 ± 0.1†	20.49 ± 2.58†

*: *P* < 0.05 (single dose compared with multiple dose); †: *P* < 0.05 (male rats compared with female rats).

Oral doses of DHA (10 mg/kg) once daily for five consecutive days did not result in a significant decrease (*P* > 0.05) in AUC_*0*-*t*_, C_max_, T_max_, MRT, CL or V_z_ of DHA in either male rats or female rats compared with values obtained after a single oral dose. Similar to the parent drug DHA, the phase I/II metabolites M1 and DHA-G did not show time-dependent pharmacokinetics. In a previous study, there were no differences in the concentrations of DHA in Vietnamese patients within the first two days of treatment with DHA. The pharmacokinetics of DHA in the acute phase, however, was significantly different from that in the convalescent phase of malaria, which suggested that the change in pharmacokinetics of DHA is related to the physiological change in malaria patients between the acute and convalescent phases of the disease [15]. There was a time-dependent decrease of DHA plasma concentrations after the same repeated doses of Artekin tablets (DHA-piperaquine) in healthy subjects. The inconsistent pharmacokinetics of DHA may be caused by different formulations of DHA, the manufacturing process, different prescriptions and ethnicity.

It is well known that remarkable decline in plasma drug concentration was observed after repeated oral dosing of QHS in humans and laboratory animals [9,20]. Auto-induction metabolism was supposed to be the underlying mechanism. The elimination of QHS drugs was reported to be mediated primarily by CYP2B6 and CYP3A4 [11,12]. Artemisinin anti-malarials moderately affect cytochrome P450 enzyme activity in healthy subjects, and the 1-hydroxymidazolam/midazolam 4-h plasma concentration ratio (CYP3A activity) was increased after 5-day treatment by QHS (2.66-fold), artemether (1.54-fold) and slightly by DHA (1.25-fold) [21]. In a population pharmacokinetics study, the activity of CYP2B6 was increased 79.7% by QHS and 19.9% by DHA [11]. It was found that QHS could induce the activity of CYP3A4 (E_max_ 3.5-fold and EC_50_ 5.9 μM) and CYP2B6 (E_max_ 1.9-fold and EC_50_ 0.6 μM) in primary human hepatocytes, whereas DHA has a small inductive potential [22]. QHS could activate nuclear receptors PXR and CAR, and result in the induction of the expression of CYP2B6, CYP3A4, and MDR1 in primary human hepatocytes and in the human intestinal cell line LS174T [23]. 50 μM artemether and arteether activated hPXR and hCAR comparable to 100 μM QHS. In contrast, DHA did not significantly activate hPXR. These previous reports showed that the time-dependent pharmacokinetics and auto-induction metabolism were different between artemisinin drugs. Even though the auto-induction metabolism was confirmed for QHS, there was limited direct evidence on whether the concentrations of DHA metabolites really increased after multiple drug administrations. In the present study, time-dependent pharmacokinetics was not found for DHA and its major phase I/II metabolites in rats, which was different from QHS [20].

On the other hand, there was significant difference (*P* < 0.05) in AUC_*0*-*t*_ and *C*_max_ between male rats and female rats in the present study. Male rats showed higher concentration levels of DHA, M1 and DHA-G with lower CL values than female rats after a single or multiple oral doses. Simpson *et al*[17] suggested that the pharmacokinetic properties of DHA were affected only by gender and body weight in malarial patients. However, another pharmacokinetic study showed that there was no significant difference (P > 0.05) in gender, which indicated that gender did not affect or change the absorption, distribution and elimination of DHA in healthy volunteers [18]. The inconsistent data resulting from gender may be caused by a different metabolism in healthy volunteers and malarial patients.

Although the validity of animal experiments to predict efficacy and safety in human has been questioned, it is generally believed that pharmacokinetic (PK) data can be extrapolated to humans reasonably well, using the appropriate PK principles [24]. Moreover, it is important to realize that humans differ from animals with regard to CYP isoform composition, expression and catalytic activities [24]. Interestingly, CYP2B has been found to be sexual dimorphic in human and rat, and CYP3A forms appear to be expressed in a sex-specific manner in rats [24]. Additionally, sex differences in UGT activity are reported relatively small and are confined to several UGTs, including UGT2B15, which shows higher activity in males, compared with females [25]. CYP2B, CYP3A and UGT enzymes can be induced in both rodent and non-rodent species, but these CYP or UGT isoforms expressed in different species show different substrate specifications. Thus, some caution should be applied when extrapolating metabolism data of DHA from animal models to humans. Further assessment of induction of CYP (particularly CYP2B and CYP3A) and UGT enzymes involved in the metabolism of DHA, is required to clarify the entire picture of species differences between humans and rats.

### Pharmacokinetics of ARS and its phase I/II metabolites

**Figure 4:**
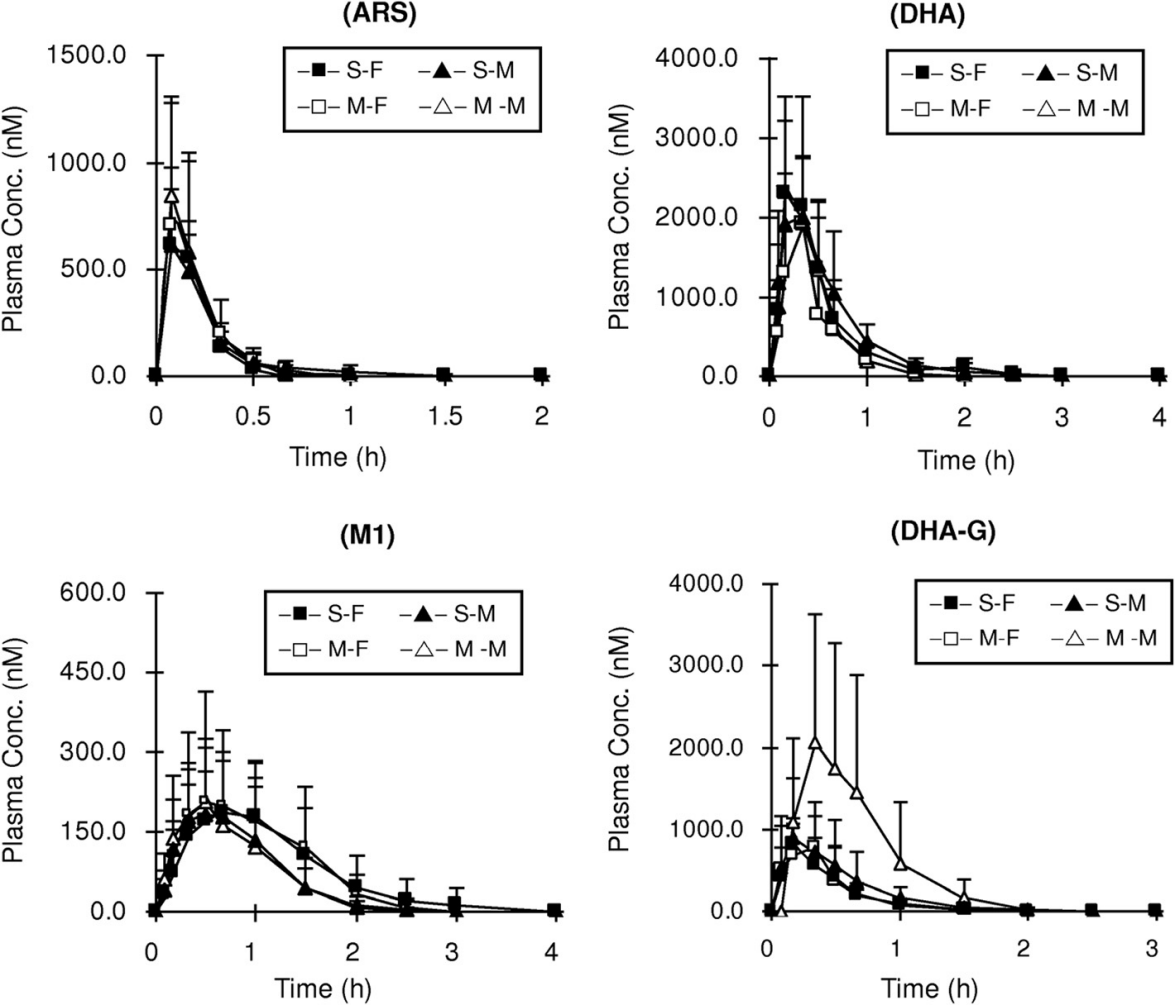
Mean (+S.D.) plasma concentration-time profiles of artesunate (ARS), dihydroartemisinin (DHA), the monohydroxylated metabolite of DHA (M1) and the glucuronide of DHA (DHA-G) in male (triangular points; S-M and M-M) and female (square points; S-F and M-F) rats (n = 6 each) following a single oral administration (solid points) or five consecutive days of oral doses (open points) of ARS (10 mg/kg).

After ARS was orally administered to rats, the parent drug ARS, the active metabolite DHA and its subsequent metabolites (M1 and DHA-G) could be detected (Figure 2). Their mean plasma concentration-time profiles are shown in Figure 4, and the pharmacokinetic parameters of ARS and its metabolites are presented in Table 2.

**Table 2:** The main pharmacokinetic parameters of artesunate (ARS) and its phase I/II metabolites (dihydroartemisinin: DHA, M1 and DHA-G) after one-day and five-day oral administrations of ARS (10 mg/kg) to male (M) and female (F) rats (mean ± s.d)

			**AUC**_**0-t**_**(nmol⋅h⋅L**^**-1**^**)**	**C**_**max**_**(nmol⋅L**^**-1**^**)**	**T**_**max**_**(h)**	**MRT (h)**	**CL/F (L⋅min**^**-1**^**⋅kg**^**-1**^**)**
One-day	ARS	M	169.45 ± 52.77	657.30 ± 201.19	0.1 (0.1-0.2)	0.3 ± 0.1	2.78 ± 0.85
		F	149.86 ± 52.36	677.70 ± 296.28	0.1 (0.1-0.2)	0.2 ± 0.04	3.15 ± 0.92
	DHA	M	1470.64 ± 695.34	2139.18 ± 737.53	0.2 (0.2-0.3)	0.6 ± 0.1*	0.34 ± 0.13
		F	1357.65 ± 564.25	2867.33 ± 982.21	0.3 (0.2-0.5)	0.6 ± 0.1	0.38 ± 0.18
	M1	M	208.79 ± 140.96	197.74 ± 135.08	0.5 (0.3-0.7)†	0.8 ± 0.2	2.74 ± 1.36
		F	280.05 ± 202.13	195.61 ± 89.26	0.8 (0.5-1.0)*†	1.0 ± 0.2	2.55 ± 1.94
	DHA-G	M	568.52 ± 442.13	925.92 ± 708.54	0.2 (0.2-0.3)	0.6 ± 0.2*	1.08 ± 0.54
		F	395.43 ± 129.49	964.72 ± 293.54	0.2 (0.2-0.5)	0.4 ± 0.1	1.21 ± 0.43
Five-day	ARS	M	188.03 ± 107.53	864.21 ± 444.42	0.1 (0.1-0.2)	0.2 ± 0.03	3.08 ± 1.78
		F	179.43 ± 111.88	861.62 ± 580.92	0.1 (0.1-0.2)	0.2 ± 0.05	3.47 ± 2.07
	DHA	M	1171.60 ± 613.77	2531.74 ± 1177.65	0.2 (0.2-0.3)	0.4 ± 0.1*	0.50 ± 0.32
		F	893.01 ± 468.66	2008.96 ± 925.29	0.3 (0.1-0.3)	0.4 ± 0.1	0.61 ± 0.32
	M1	M	203.02 ± 226.62	219.10 ± 195.25	0.4 (0.3-0.5)	0.6 ± 0.2†	9.50 ± 12.08
		F	282.03 ± 170.42	216.74 ± 100.50	0.5 (0.3-0.7)*	0.9 ± 0.2†	2.11 ± 1.31
	DHA-G	M	1105.48 ± 986.09	2396.47 ± 1827.67	0.2 (0.2-0.3)	0.4 ± 0.1*	0.82 ± 0.72
		F	406.74 ± 135.55	1025.37 ± 484.94	0.2 (0.1-0.3)	0.4 ± 0.2	1.20 ± 0.48

*: *P* < 0.05 (single dose compared with multiple dose); †: *P* < 0.05 (male rats compared with female rats).

Similar to DHA, oral doses of ARS (10 mg/kg) to male or female rats once daily for five consecutive days did not result in a significant change (*P* > 0.05) in AUC_*0*-*t*_ or C_max_ of ARS and its major metabolites, including DHA and its subsequent metabolites (M1 and DHA-G). Other 5-day oral dosing studies of ARS did not show time-dependent pharmacokinetics of ARS and its metabolite DHA in healthy subjects [14] and rats [26].

The effectiveness of ARS has been mostly attributed to its rapid (t_1/2_, 2-3 min) and extensive hydrolysis to DHA [5]. Unexpectedly, sex difference was not observed for ARS and its metabolites in rats after a single oral dose of ARS. The gender difference in AUC and C_max_ by treatment with DHA but not ARS indicated that the absorption process may play an important role. Even though the species difference need to be carefully considered when extrapolating metabolism data of DHA from animal models to humans, the effect of the formulation of DHA tablets on the gender difference in pharmacokinetics may still be clinically relevant.

## Conclusions

The results gave the direct evidence for the absence of auto-induction phase I/II metabolism of DHA and ARS. The sex effect existed for DHA but not for ARS, which could be caused by the sex-specific differences in absorption of DHA. From the point of auto-induction metabolism, DHA seems to be a more promising anti-malarials than its parent drug QHS and its derivative artemether. The present study also showed the inductive capacity is different among QHS drugs, which is important when selecting drugs to be used in anti-malarial combination therapy such that the potential for drug-drug interactions can be minimized.

## Competing interests

The authors declare that they have no competing interests.

## Authors’ contributions

FZ performed the experiments and analysed the data. FD and XL helped in performing the experiments, and FD contributed in review of manuscript. JX designed the experiments, analysed the data, and wrote the paper. All authors read and approved the final manuscript.
